# *Brucella* Omp25 Upregulates miR-155, miR-21-5p, and miR-23b to Inhibit Interleukin-12 Production *via* Modulation of Programmed Death-1 Signaling in Human Monocyte/Macrophages

**DOI:** 10.3389/fimmu.2017.00708

**Published:** 2017-06-26

**Authors:** Beibei Cui, Wenli Liu, Xiaoya Wang, Yu Chen, Qian Du, Xiaomin Zhao, Hai Zhang, Shan-Lu Liu, Dewen Tong, Yong Huang

**Affiliations:** ^1^College of Veterinary Medicine, Northwest A&F University, Yangling, China; ^2^School Hospital, Northwest A&F University, Yangling, China; ^3^Laboratory Animal Center, Fourth Military Medical University, Xi’an, China; ^4^Center for Retrovirus Research, Department of Veterinary Biosciences, Department of Microbial Infection and Immunity, The Ohio State University, Columbus, OH, United States

**Keywords:** cytokine, Omp25, miRNA, macrophage, signaling

## Abstract

*Brucella* spp. infection results in compromised Type1 (Th1) cellular immune response. Several reports have described an immunomodulatory function for *Brucella* major outer membrane protein Omp25. However, the mechanism by which Omp25 modulates macrophage dysfunction has not been defined. Herein, we reported that Omp25-deficient mutant of *Brucella suis* exhibited an enhanced ability to induce interleukin (IL)-12 whereas ectopic expression of Omp25 protein inhibited TLR agonists-induced IL-12 p70 production through suppression of both IL-12 p40 and p35 subunit expression in THP-1 cells. In addition, Omp25 significantly upregulated miR-155, -23b and -21-5p, as well as the immunomodulator molecule programmed death-1 (PD-1) in monocyte/macrophages. The upregulation of miR-155 and -23b correlated temporally with decreased TAB2 levels, IκB phosphorylation and IL-12 p40 levels by targeting TAB2 and *il12B* 3′ untranslated region (UTR), respectively, while miR-21-5p increase directly led to the reduction of lipopolysaccharide (LPS)/R848-induced IL-12 p35 protein by targeting *il12A* 3′UTR. Consistent with this finding, reduction of miR-155 and -23b attenuated the inhibitory effects of Omp25 on LPS/R848-induced IL-12 p40 expression at both transcriptional and posttranscriptional levels, while reduction of miR-21-5p attenuated the inhibitory effects of Omp25 on LPS/R848-induced IL-12 p35 expression at the posttranscriptional level, together significantly enhanced IL-12 p70 production upon LPS/R848 stimulation. We also found that blocking PD-1 signaling decreased the expression of miR-155, -23b and -21-5p induced by Omp25 and enhanced IL-12 production in monocyte/macrophages. Altogether, these data demonstrate that *Brucella* Omp25 induces miR-155, -23b and -21-5p to negatively regulate IL-12 production at both transcriptional and posttranscriptional levels *via* regulation of PD-1 signaling, which provides an entirely new mechanism underlying monocyte/macrophages dysfunction during *Brucella* spp. infection.

## Introduction

*Brucella* spp. is an intracellular pathogen that resides mainly in monocyte/macrophages and causes disease in humans and livestocks (1–3). Resistance to *Brucella* spp. relies on cell-mediated immunity, which involves the activation of antigen-presenting cells (macrophages and dendritic cells) and the subsequent activation of antigen-specific CD4^+^ and CD8^+^ T cells (4, 5). In this process, the production of T helper type 1 (Th1) cytokines and an adequate Th1 immune response are critical for the clearance of *Brucella* infection (6, 7). Studies on experimental and human brucellosis indicate that interleukin (IL)-12 is the principal cytokine active against *Brucella* infection (8–10). However, *Brucella* has evolutionarily developed diverse evasion strategies to interfere the host’s innate and adaptive immunity in order to establish a long-term infection (11). Disturbances of IL-12 production and Th1 response have been described in patients with chronic brucellosis and are associated with poor outcome, but relative mechanisms remain largely unknown (5, 12).

The major outer membrane protein Omp25 is highly conserved across *Brucella* species, biovars, and strains (13). Several reports have shown that administration of Omp25 DNA vaccine or recombinant Omp25 is protective against the virulent *Brucella melitensis* or *Brucella abortus* challenge in mice (14, 15), which makes Omp25 as a viable vaccine target. On the other hand, however, Omp25 has also been reported to be involved in virulence of *B. melitensis, B. abortus*, and *Brucella ovis* (16). *Brucella* species lacking Omp25 have been shown to be attenuated in mice as well as cattle (17, 18). Live *Brucella* spp. fail to induce tumor necrosis factor alpha (TNF-α) (19), whereas Omp25 null mutants of *Brucella suis* (Δ*omp25 B. suis*) exhibit the ability to activate human macrophages to secrete TNF-α, suggesting that Omp25 is involved in inhibition of TNF-α production during infection of human macrophages (20). Similarly, the presence of Omp25 in wild-type (WT) *Brucella* spp. is correlated with the unusual absence of TNF-α and IL-12 in human dendritic cells, which prevents human dendritic cell maturation and antigen presentation (5). Whereas treatment of *Brucella*-susceptible mice with IL-12 increases primary and secondary immunity (8), the molecular mechanism(s) by which *Brucella* Omp25 inhibits IL-12 production has not been defined.

In the present work, we evaluated the effects of Omp25 on the Toll-like receptor 4 and 7/8 agonist lipopolysaccharide (LPS)/R848-induced IL-12 expression in monocyte/macrophages (M/MΦs). We found that ectopic expression of Omp25 protein inhibited TLR agonists-induced IL-12 p70 production through suppression of both IL-12 p40 and p35 subunit expression in THP-1 cells. MiR-155, -23b and -21-5p were upregulated in Omp25-expressing cells, as well as in WT *B. suis*-infected human M/MΦs, and were identified to be involved in the inhibition of LPS/R848-induced IL-12 through inhibiting the expression of IL-12 p35 and p40 at transcriptional and posttranscriptional levels, respectively. We also found that Omp25 induced programmed death-1 (PD-1) expression that is inversely associated with IL-12 production in human M/MΦs. Blocking of PD-1 signaling decreased miR-155, miR-21-5p and miR-23 levels and enhanced IL-12 production.

## Materials and Methods

### Cell Isolation and Culture

Peripheral blood mononuclear cells (PBMCs) were prepared from healthy adult donors by centrifugation over Ficoll-Histopaque (Sigma, St. Louis, MO, USA) as described (21). All the donors had signed an informed consent before their blood was used in this study. M/MΦs cells were enriched from PBMCs using Ficoll-Percoll gradients (GE Healthcare) and further purified by anti-CD14 magnetic beads with column purification according to the manufacturer’s instructions (Miltenyi Biotec; purity of cells >95%). The enriched cells were cultured in RPMI 1640 (Invitrogen) supplemented with 10% fetal bovine serum (FBS, Hyclone).

The human monocytic cell line THP-1 and HEK-293 cells were purchased from American Type Culture Collection (ATCC, Manassas, VA, USA). THP-1 were maintained in RPMI 1640 medium and HEK-293 cells were maintained in Dulbecco’s minimum essential medium, both supplemented with 10% FBS at 37°C with 5% CO_2_.

### Bacterial Strains and Recombinant Adenovirus Preparation

*Brucella suis* 1330 (ATCC 23444) and its derived mutants were cultured in tryptic soy broth at 37°C. Omp25-deficient mutant (Δ*omp25 B. suis*) were obtained using suicide plasmid PCVD442 carrying *omp25* gene interrupted by kanamycin resistance gene as previously described (20). In this study, all live *Brucella* experiments were performed in biosafety level 3 facilities according to standard procedures. Either deleted *omp25* gene or expressed Omp25 protein was omp25, not omp25b, omp25c, omp23d, or omp22 (22). The native *omp25* gene (GenBank No. U39397.1) was amplified by PCR from *B. suis* 1330 using *omp25* specific Primers with Flag tag encoding sequence (Table S1 in Supplementary Material), then *omp25* gene was inserted into the vector pShuttle-CMV (Clontech) to construct the adenoviral shuttle vector pShuttle-CMV-Omp25. The linearized pShuttle-CMV-Omp25 and pShuttle-CMV were transformed into BJ5183-AD-1 by electroporation to recombine with pAd-Easy-1, followed by amplification and extraction of the recombinant vector named pAd-Omp25 and pAd-Blank. The recombinant adenovirus named recombinant adenoviruses expressing Omp25 protein (rAd-Omp25) and rAd-Blank were obtained by transferring the linearized pAd-Omp25 and pAd-Blank to HEK-293 cells. The titer of rAd-Omp25 and rAd-Blank were measured by the method of TCID_50_ as described in previous studies (23).

### *Brucella* Infection and Intracellular Survival Assay

Human THP-1 cells were cultured with 100 nM 1,25-dihydroxyvitamin D3 (VD3, Sigma) for 72 h before *Brucella* infection as previously described (24). THP-1 cells and human M/MΦs were infected with *B. suis* as previously described (24, 25). Briefly, 1 × 10^6^ cells/ml or 2 × 10^5^ cells/ml of VD3-treated THP-1 cells or human M/MΦs were cultured in RPMI 1640 medium with 10% heat-inactivated FBS and infected with *B. suis* 1330 or mutated *B. suis* strains at a multiplicity of infection of 50 for 1 h at 37°C in a 5% CO_2_ atmosphere. At the end of the incubation time (time 0 p.i.), cells were washed three times with sterile PBS to remove uninternalized bacteria, and then cells were cultured in standard medium with 50 µg/ml of gentamicin (Sigma) and 50 µg/ml of streptomycin (Sigma) to kill remaining extracellular bacteria.

To monitor *Brucella* survival in cells, infected cells (5 × 10^5^ cells/well) were washed with PBS and lysed in 0.2% Triton X-100 (Sigma). Serial dilutions of lysates were rapidly plated on tryptic soy agar plates to count and calculate the number of intracellular viable bacteria in CFU per well.

### MiRNA Target Predictions

Sequences of predicted mature human miRNAs were obtained from miRBase.^1^ *Il12A* and *il12B* sequences were from the National Center for Biotechnology Information Nucleotide database.^2^ MiRNAs predicted to bind to 3′ untranslated regions (UTRs) of *il12A* and *il12B* genes were selected by target analysis through the prediction algorithms Targetscan human and RNAhybrid.

### Quantitative PCR

MicroRNAs and IL-12 p40 or p35 subunit mRNA was quantified by quantitative polymerase chain reaction (Q-PCR). Total cellular RNA was isolated by TRIZOL according to the manufactory’s protocol (26). RNA concentration and purity were measured using a NanoDrop spectrophotometer (Thermo), and equal amounts of RNA (10 ng for miRNA or mRNA) were used for Q-PCR analysis. MiRNA and mRNA levels were detected by SYBR Green kit using Bio-Rad IQ5 Real-time system and calculated using RNU6B and β-actin as endogenous control, respectively, following the 2^−ΔΔCt^ method. Primers used in this assay were presented in Table S1 in Supplementary Material.

### Western Blotting Analysis

Cells were lysed in RIPA plus protease/phosphorylase inhibitors (Sigma) on ice. The cytosol and nuclear fraction were isolated using NE-PER Nuclear and Cytoplasmic Extraction Reagents according to the manufactory’s instruction (Thermo). Protein from each extract were separated on 4–12% SDS-PAGE and transferred to polyvinyl difluoride membranes (Millipore). After blocking with TBS plus 0.05% Tween 20 containing 5% non-fat milk for 1 h, the membranes were incubated with specific primary antibodies at 4°C overnight. Primary antibodies include anti-IL-12 p40/p35 (Santa Cruz), anti-TAB2, anti-phospho-IκB, anti-IκB, anti-p65, anti-TRAF6, anti-IRAK1, anti-IRAK2, anti-Histone H3 (Cell Signaling Technology), anti-FLAG M2 (Sigma), anti-Omp25, and anti-β-actin (Tianjin Sungene Biotech) antibodies. HRP-conjugated anti-mouse IgG or anti-rabbit IgG (Santa Cruz) were used as secondary antibodies. Pierce^®^ Fast Western Blot Kit, ECL Substrate (Thermo) was used for chemiluminescent detection.

### Luciferase Reporter Assays

Human *il12A* or *il12B* promoter sequence was cloned into pGL3 basic vector (Promega) to construct the promoter report vector pGL-il12A, pGL-il12B using specific primers (Table S1 in Supplementary Material). The pGL3 basic vector served as a control. THP-1 cells were transfected with a mixture of pGL-il12A, pGL-il12B, or NF-κB activity reporter plasmid and pRL-TK-renilla-luciferase plasmid (Promega, for normalization) using Amaxa nucleofection technology (Human monocyte Nucleofector Kit, Lonza). Cells were then infected with rAd-Blank or rAd-Omp25. At 24 h post infection, luciferase activities were determined *via* Dual-Luciferase Reporter Assay System (Promega) according to the manufacturer’s instructions.

The full-length 3′ UTR of the human *il12A* gene (GenBank accession no. NM_000882) or *il12B* gene (GenBank accession no. NM_002187) was amplified from human THP-1 cDNA using specific primers (Table S1 in Supplementary Material) and cloned into pMIR-REPORT Luciferase vector (Ambion). The segment (base pairs 984–1450) of the *il12A* 3′UTR containing the mutated miR-21-5p target sequence (ATAAGCT to TATTCGA) and the segment (base pairs 1030–2347) of the *il12B* 3′UTR containing the mutated miR-23b target sequence (AATGTGA to TTACACT) were also cloned into the pMIR-REPORT Luciferase vector (Ambion). The primers for mutants were also showed in Table S1 in Supplementary Material. HEK-293 cells were transfected with the indicated luciferase reporter plasmid (500 ng), pRL-TK-Renilla-luciferase plasmid (100 ng), and the indicated miRNA mimics (final concentration, 10 or 50 nM) by Lipofectamine 2000 (Invitrogen). At 48 h post transfection, cellular extracts were prepared and measured luciferase activities by the Dual-Luciferase Reporter Assay System (Promega) according to the manufacturer’s instructions.

### Flow Cytometry and Cytokine Detection

Cell surface molecules and IL-12 expression in monocyte/macrophages were determined by flow cytometric analysis as described previously (27, 28). Briefly, 1 × 10^6^ cells were infected with WT *B. suis*, Δ*omp25 B. suis*, rAd-Omp25 or rAd-Blank for 24 or 48 h to harvest the cells for immune staining. Cells were first stained for cell surface molecules using APC-anti-human CD14 Ab and PE-anti-PD-1 or PE-anti-PD-L1 Ab (Biolegend) in staining buffer and were then treated with fixation and permeabilization buffer (Biolegend) for intracellular staining according to the manufacturer’s instruction. Stained cells were analyzed by flow cytometry (BD Accuri C6) and further analyzed on flowJo V7.6 software as previous (29). Commercial IL-12 p70 ELISA kits (Biolegend) were used to measure the levels of IL-12 p70 in cell-free culture supernatants according to the manufacturer’s instructions.

### Statistical Analysis

All experiments were performed at least three times, and results are representative of three independent experiments. Prism software (GraphPad Software) was used for statistical analysis. Data were presented as means ± SEM. Statistical comparisons of the results were made using ANOVA followed by Bonferroni *post hoc* test or performed by unpaired Student’s *t*-test. Pearson’s correlation coefficients were calculated to examine the correlations between PD-1^+^ or PD-L1^+^ cells with IL-12^+^ cells. *P* < 0.05 or less was considered significant.

## Results

### Omp25-Deficient Mutant of *B. suis* Exhibit an Enhanced Ability to Induce IL-12 in Infected THP-1 Cells

**Figure 1:**
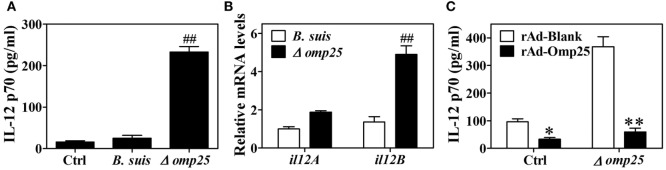
The Δ*omp25 Brucella suis* mutant induces interleukin (IL)-12 production in human THP-1 cells. **(A)** THP-1 cells were infected with wild-type (WT) *B. suis*, Omp25 mutant (Δ*omp25*) or were uninfected (Ctrl) as described in Section “Materials and Methods,” and cells were cultured for 24 h in complete culture medium. The concentrations of IL-12 p70 in cell supernatants were determined by ELISA. **(B)** THP-1 cells were infected as in **(A)** and cultured for 6 h, followed by quantitative polymerase chain reaction detection of *il12A* (p35) and *il12B* (p40) mRNAs levels in THP-1 cells. **(C)** THP-1 cells were infected with 100 multiplicity of infection of recombinant adenoviruses expressing Omp25 protein (rAd-Omp25) or rAd-Blank for 24 h and then infected with Δ*omp25* for another 24 h or uninfected (Ctrl), followed by ELISA detection of IL-12 p70 in cell supernatants. Each value represents means ± SEMs of three independent experiments. ^##^*P* < 0.01 versus WT *B. suis*-infected cells. *P < 0.05, **P < 0.01 versus rAd-Blank-infected cells in same treatment.

Omp25 is an outer membrane protein on the surface of *Brucella*, and Omp25-deficient mutants have been shown to exhibit enhanced ability to secret TNF-α compared with WT *Brucella* (5, 20). Since IL-12 is also an important cytokine for clearance of intracellular pathogens, we investigated the effects of Omp25 on the expression of IL-12. We first confirmed the presence of Omp25 in WT *B. suis-*infected cells and the absence of Omp25 in the corresponding Omp25-deficient mutant (Δ*omp25 B. suis*)-infected cells (Figure S1 in Supplementary Material). Next, we observed the intracellular survival of WT *B. suis and* Δ*omp25 B. suis*, and compared the production of IL-12 in WT *B. suis-* and Δ*omp25 B. suis-*infected human THP-1 cells. As would be expected, the numbers of intracellular viable bacteria were similar for WT and Δ*omp25 B. suis* infection (Figure S2 in Supplementary Material), but Δ*omp25 B. suis*-infected cells produced more IL-12, ranging from 210 to 256 pg/ml, than that of WT (Figure 1A, *P* < 0.01). Similarly, the level of IL-12 p40 mRNA was higher in the Δ*omp25 B. suis*-infected cells than that in WT *B. suis*-infected cells (Figure 1B), suggesting that the absence of Omp25 might improve *B. suis*’s ability to induce IL-12. To further confirm whether Omp25 play a key role in inhibition of IL-12 production, THP-1 cells were infected with recombinant adenoviruses rAd-Omp25 to express Omp25, and then infection of Δ*omp25 B. suis* and measured the production of IL-12. Results showed that the IL-12 p70 production of rAd-Omp25-infected cells was significantly lower than that of control blank adenoviruses (rAd-Blank)-infected cells upon Δ*omp25 B. suis* infection (Figure 1C), in which cells were infected with rAd-Omp25 to complement Omp25 (Figure S1 in Supplementary Material). These results indicated that the presence of Omp25 inhibited *B. suis*’s ability to induce IL-12.

### Omp25 Inhibits LPS/R848-Induced IL-12 Production in THP-1 Cells

**Figure 2:**
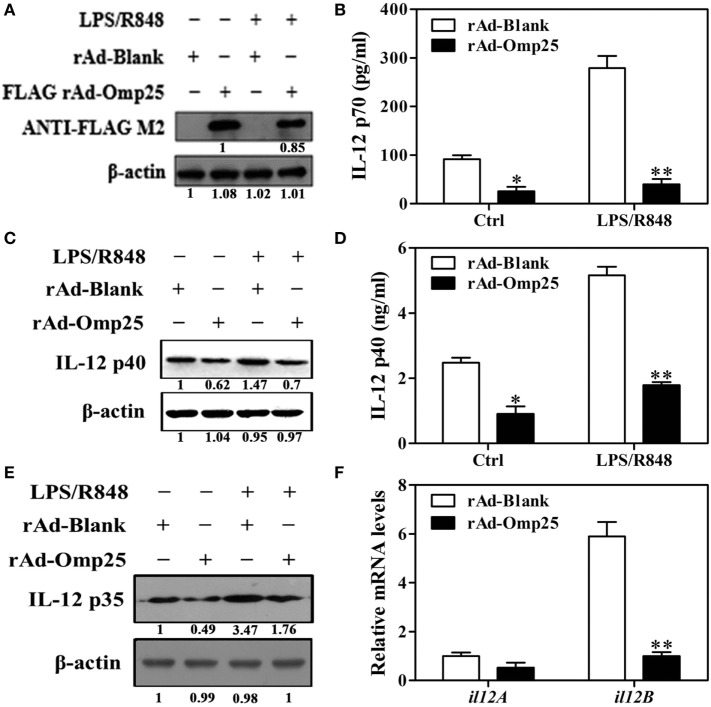
Omp25 inhibits lipopolysaccharide (LPS)/R848-induced interleukin (IL)-12 expression in THP-1 cells. **(A)** Expression of Omp25 in THP-1 cells infected with recombinant adenoviruses expressing Omp25 protein (rAd-Omp25) or rAd-Blank. THP-1 cells were infected with 100 multiplicity of infection of rAd-Omp25 or rAd-Blank for 24 h and then treated with LPS/R848 or PBS for 24 h, followed by western blot detection. **(B)** Omp25 inhibits LPS/R848-induced IL-12 p70 secretion in THP-1 cells. Cells were infected and treated as in **(A)**, and supernatant IL-12 p70 were measured by ELISA. **(C–E)** Omp25 inhibits LPS/R848-induced IL-12 p35 and p40 in THP-1 cells. Cells were infected and treated as in **(A)**; and the levels of IL-12 p40 **(C,D)** or IL-12 p35 **(E)** were detected by ELISA and western blot. **(F)** Omp25 decreases *il12B* (p40) mRNAs in THP-1 cells. THP-1 cells were infected rAd-Omp25 or rAd-Blank for 24 h and stimulated by LPS/R848 for 6 h followed by quantitative polymerase chain reaction detection of *il12A* and *il12B* mRNA levels. The results are means ± SEMs of three independent experiments. *P < 0.05, **P < 0.01 versus rAd-Blank-infected cells in same treatment.

To investigate the direct effects of Omp25 protein on IL-12 p70 production in macrophage, we examined the LPS/R848-induced IL-12 p70 production in THP-1 cells infected with rAd-Omp25 or control blank adenoviruses (rAd-Blank). We found that Omp25 was stably expressed in the THP-1 cells infected with rAd-Omp25 (Figure 2A), which inhibited IL-12 p70 production induced either by LPS/R848 or recombinant adenoviruses themselves (Figure 2B). We also observed that the expression of IL-12 p35 and p40 subunit induced by LPS/R848 was lower in rAd-Omp25-infected cells compared to that in rAd-Blank-infected cells (Figures 2C–E). Similarly, upon LPS/R848 stimulation, the *il12B* (p40) mRNA level of rAd-Omp25-infected cells was lower than that of rAd-Blank-infected cells, but *il12A* (p35) mRNA level was not significantly difference between these two cells (Figure 2F). These results indicated that Omp25 can directly inhibit the production of LPS/R848-induced IL-12 p70 through suppressing p40 subunit expression at both protein and mRNA levels, yet suppressing p35 subunit only at protein level in THP-1 cells. Our data also suggested that the expression of both p35 and p40 subunits are regulated at the posttranscriptional level in Omp25-expressed cells.

### Omp25 Inhibits the Activation of NF-κB Pathway to Attenuate IL-12 p40 but Not IL-12 p35 Transcription

**Figure 3:**
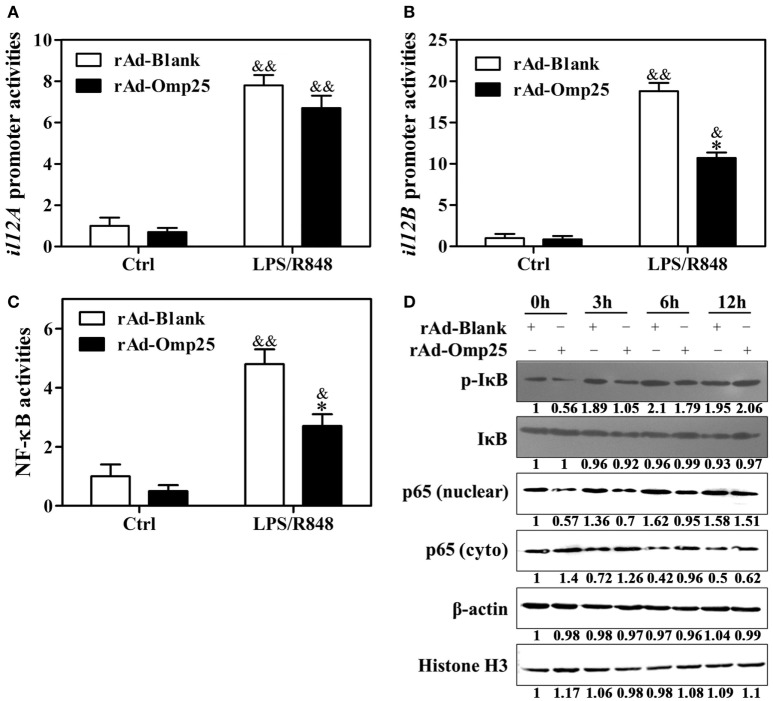
Omp25 inhibits the activation of NF-κB pathway to attenuate interleukin-12 p40 transcription. **(A–C)** THP-1 cells were infected with rAd-Blank or recombinant adenoviruses expressing Omp25 protein (rAd-Omp25) for 24 h and then treated with lipopolysaccharide (LPS)/R848 for another 24 h. The promoter relative activities of *il12A* **(A)** and *il12B* **(B)** and the transcriptional relative activities of NF-κB **(C)** were measured. The results are means ± SEMs of three independent experiments. *P < 0.05 versus rAd-Blank infected cells; ^&^*P* < 0.05, ^&&^*P* < 0.01 versus control (Ctrl). **(D)** THP-1 cells were infected with 100 multiplicity of infection rAd-Blank or rAd-Omp25 for 24 h. Western blotting was used to examine the distribution of cytoplasmic and nuclear p65 at indicated times following treatment with LPS/R848 for another 0, 3, 6, and 12 h; similar treatments applied to p-IκB and IκB.

To investigate the effects of Omp25 on the transcription of p35 and p40 subunit, we examined the promoter activities of *il12A* and *il12B* and NF-κB transcriptional activities in rAd-Omp25-infected cells and rAd-Blank-infected cells. Omp25 expression did not appear to affect IL-12 p35 transcription (Figure 3A), but did inhibit IL-12 p40 transcription (Figure 3B). Upon LPS/R848 treatment, the transcriptional activities of NF-κB were lower in rAd-Omp25-infected cells than that in rAd-Blank-infected cells (Figure 3C). In line with the changes of NF-κB activities, the phosphorylation levels of IκB and the levels of nuclear NF-κB p65 unit were lower in rAd-Omp25-infected cells than that in rAd-Blank-infected cells at 0, 3, and 6 h after LPS/R848 treatment (Figure 3D). Given the key roles of NF-κB p65 unit in the transcription of *il12B*, these results further demonstrated that Omp25 inhibits the expression of p40 subunit but not p35 subunit at the transcriptional level.

### Omp25 Upregulates miR-21-5p, miR-23b, and miR-155 in THP-1 Cells

**Figure 4:**
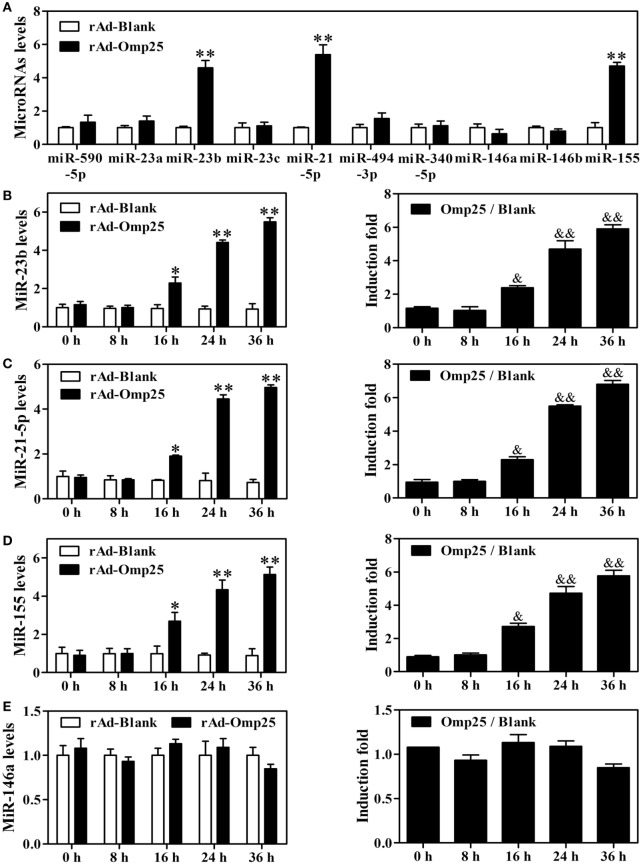
Omp25 upregulates miR-155, miR-23b and miR-21-5p expression in THP-1 cells. **(A)** Effects of Omp25 expression on the expression profiles of microRNAs in THP-1 cells. THP-1 cells were infected with recombinant adenoviruses expressing Omp25 protein (rAd-Omp25) or rAd-Blank for 24 h, and indicated miRNAs were measured by Quantitative PCR and normalized to the expression of RNU6B in each sample. **(B–E)** The relative expression of miR-23b **(B)**, miR-21-5p **(C)**, miR-155 **(D)** and miR-146a **(E)** levels were analyzed by quantitative polymerase chain reaction. Omp25/Blank represents the fold changes in RNA levels as calculated by comparing the value of rAd-Omp25-infected cells to that of rAd-Blank-infected cells in parallel. The results are means ± SEMs of three independent experiments. *P < 0.05, **P < 0.01 versus rAd-Blank-infected cells; ^&^*P* < 0.05, ^&&^*P* < 0.01 versus 0 h uninfected cells.

To investigate the potential role of microRNAs in regulating IL-12 p40 and p35 in Omp25-expressed cells, we used multiple prediction algorithms, including Targetscan human, miRanda, and RNA hybrid, to search for conserved microRNAs whose target sites exist in the *il12A* and *il12B* 3′ UTRs of human and other mammals. In total, seven miRNA–mRNA target duplexes were predicted by these algorithms (Figure S3 in Supplementary Material), including miR-590-5p, miR-21-5p, miR-340-5p, miR-23a, miR-23b, miR-23c, and miR-494-3p. To determine which miRNAs might be regulated by Omp25 in THP-1 cells, we examined the expression profile of these miRNAs, as well as some other miRNAs that have been reported to regulate NF-κB signaling, such as miR-146a, miR-146b, and miR-155. Q-PCR profiling of 10 miRNAs revealed that miR-21-5p, miR-23b and miR-155 were higher in rAd-Omp25-infected cells than that in control rAd-Blank-infected cells (Figure 4A). This result suggested that these three miRNAs might be involved in the regulation of IL-12 expression in Omp25-expressed-THP-1 cells.

To identify the regulatory roles of miR-21-5p, miR-23b, and miR-155 in the inhibitory effects of Omp25 in THP-1 cells, we first examined the characteristics of miR-21-5p, miR-23b and miR-155 in THP-1 cells after expression of Omp25. A detailed time-course experiment showed that miR-23b, miR-155 and miR-21-5p levels were significantly increased at 16, 24 and 36 h as shown in the Figures 4B–D left panels after infecting rAd-Omp25, respectively. To further understand the kinetics of these miRNAs in the process of Omp25 expression, the fold changes of miRNAs expression were calculated by comparing the value of Omp25-expressing cells to that of control cells without Omp25 in parallel. MiR-23b showed an average increase of 2.4-fold after 16 h, rising to 5.9-fold by 36 h (Figure 4B right panel). MiR-21-5p expression showed increases of 2.3-fold by 16 h of infection and further increased at 24 and 36 h after infection (Figure 4C right panel). MiR-155 increased 2.7-fold by 16 h of infection and further increased by 4.72-fold and 5.77-fold at 24 and 36 h, respectively (Figure 4D right panel). However, no significant change was observed for the expression of miR-146a, which served as a negative control (Figure 4E). These results confirmed that the expressions of miR-21-5p, miR-23b and miR-155 are induced by Omp25, consistent with the original screen data shown in Figure 4A.

### Downregulation of miR-23b, miR-21-5p, and miR-155 Blocks Omp25 Inhibitory Effect on LPS/R848-Induced IL-12 p70 Production

**Figure 5:**
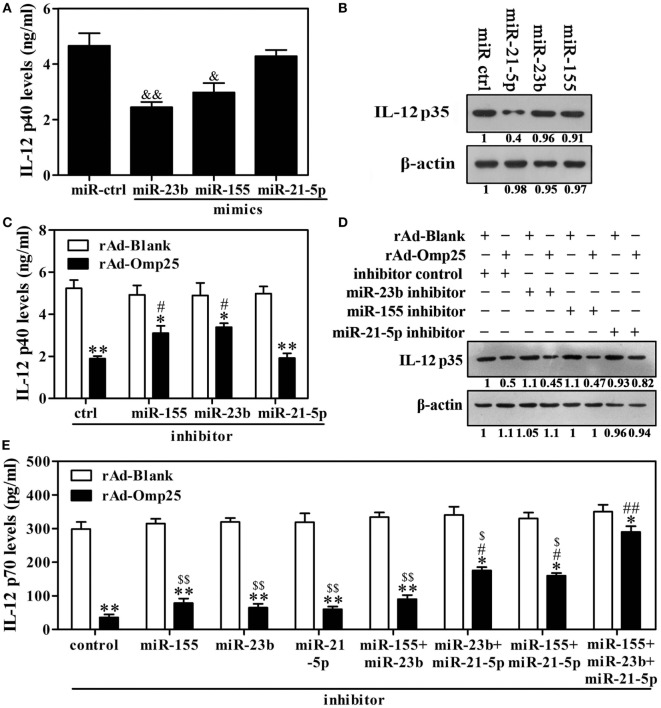
Downregulation of miR-23b and miR-21-5p partly blocks Omp25’s ability to inhibit interleukin (IL)-12 p70 that is induced by lipopolysaccharide (LPS)/R848. **(A,B)** THP-1 cells were transfected with control mimics (miR ctrl), miR-21-5p mimics, miR-23b mimics or miR-155 mimics and then treated with LPS/R848 for 24 h, the IL-12 p40 and IL-12 p35 levels were examined by ELISA **(A)** and Western blot **(B)**, respectively. **(C,D)** THP-1 cells were infected with recombinant adenoviruses expressing Omp25 protein (rAd-Omp25) or rAd-Blank and transfected with miR-21-5p inhibitor, miR-23b inhibitor, miR-155 inhibitor, or inhibitor control for 24 h and then were treated with LPS/R848 for another 24 h. The IL-12 p40 or IL-12 p35 levels were examined by ELISA **(C)** or Western blot **(D)**. **(E)** THP-1 cells were infected with rAd-Omp25 or rAd-Blank and transfected with miR-23b inhibitor, miR-21-5p inhibitor, or miR-155 inhibitor, respectively, or a mixture of two inhibitors, or a mixture of three inhibitors for 24 h, then treated with LPS/R848 for another 24 h, and the supernatants were harvested to measure IL-12 p70 secretion by ELISA. The results are means ± SEMs of three independent experiments. ^&^*P* < 0.05, ^&&^*P* < 0.01 versus miR ctrl; *P < 0.05, **P < 0.01 versus rAd-Blank-infected cells; ^#^*P* < 0.05, ^##^*P* < 0.01 versus rAd-Omp25-infected cells with inhibitor control;^$^ *P* < 0.05, ^$$^*P* < 0.01 versus rAd-Omp25-infected cells with a mixture of miR-21-5p, miR-23b and miR-155 inhibitor.

Given above results showing that Omp25 inhibits LPS/R848-induced IL-12 expression and upregulates miR-21-5p, miR-23b and miR-155 in THP-1 cells, we hypothesized that miR-21-5p, miR-23b and miR-155 likely played important roles in the Omp25 inhibition of LPS/R848-induced IL-12 p70 production. To test this hypothesis, we transfected cells with miR-21-5p mimics, miR-23b mimics or miR-155 mimics and stimulated the transfected cells with LPS/R848 for 24 h. IL-12 p40 was significantly decreased in the cells transfected with miR-23b mimics or miR-155 mimics, but not in the miR-21-5p mimics-transfected cells (Figure 5A). IL-12 p35 was significantly decreased in the cells transfected with miR-21-5p mimics, but not in the miR-23b or miR-155 mimics-transfected cells (Figure 5B). After treatment with miR-21-5p, miR-23b or miR-155 inhibitors, the expression of the respective miRNAs was downregulated over 2.5-fold, compared with control-transfected cells (Figure S4 in Supplementary Material). Inhibition of miR-23b or miR-155 induction suppressed the Omp25-induced reduction of IL-12 p40, while having no discernible effect on the Omp25-induced reduction of IL-12 p35 (Figures 5C,D). Inhibition of miR-21-5p induction suppressed the Omp25-induced reduction of IL-12 p35, while having no discernible effect on the Omp25-induced reduction of IL-12 p40 (Figures 5C,D). Consequently, inhibition of miR-21-5p, miR-23b or miR-155 induction did not significantly suppress the Omp25-induced reduction of IL-12 p70, but inhibition of both miR-21-5p and miR-23b induction, or inhibition of both miR-21-5p and miR-155 induction, significantly improved LPS/R848-induced IL-12 p70 production in the rAd-Omp25-infected THP-1 cells (Figure 5E). Notably, in cells transfected with a mix of miR-21-5p, miR-23b and miR-155 inhibitors, the inhibitory effects of Omp25 on LPS/R848-induced IL-12 p70 production were further attenuated, resulting in a level of IL-12 p70 that was higher than other inhibitor-treated cell after rAd-Omp25 infection (Figure 5E). These results demonstrated that downregulation of miR-21-5p, miR-23b, and miR-155 can block the inhibitory effects of Omp25 on LPS/R848-induced IL-12 p70.

### Omp25-Induced miR-21-5p and miR-23b Inhibit the Expression of IL-12 p35 and p40 in the Posttranscriptional Level, Respectively

Inhibition of miR-21-5p and -23b attenuates the inhibition effects of Omp25 on LPS/R848-induced IL-12 p35 and p40 expression, respectively. To confirm the possibility that IL-12 p35 and p40 were regulated posttranscriptionally by these Omp25-inducible miRNAs, we constructed reporter plasmids encoding the complete WT 3′UTR of the human *il12A* and *il12B* mRNA downstream of the firefly luciferase gene (*il12A* or *il12B* WT-3′UTR); parallel plasmids containing mismatches in the predicted miR-21-5p or -23b binding sites (miR-21-5p or 23b MT-3′UTR) of the 3′UTR region were also made (Figure S5A in Supplementary Material). In HEK-293 cells transfected with the reporter plasmids together with the indicated miRNA mimics and an internal control, pRL-TK-Renilla-luciferase, we observed that miR-21-5p and miR-23b reduced the expression of luciferase from *il12A* and *il12B* WT-3′UTR compared with either control miRNA mimics transfections (Figure S5B in Supplementary Material). However, miR-21-5p and miR-23b did not reduce luciferase expression from their respective MT-3′UTR plasmids containing specific mutations in their cognate binding sites (Figure S5B in Supplementary Material). These experiments validated the regulatory potential of miR-21-5p and miR-23b *via* binding with the *il12A* and *il12B* WT-3′UTR, respectively, and further confirmed that Omp25-induced miR-21-5p and miR-23b inhibit the expression of IL-12 p35 and p40 induced by LPS/R848 at the posttranscriptional level, respectively.

### Omp25-Induced miR-155 Expression Inhibits the Transcription of *il12B via* Targeting to TAB2

**Figure 6:**
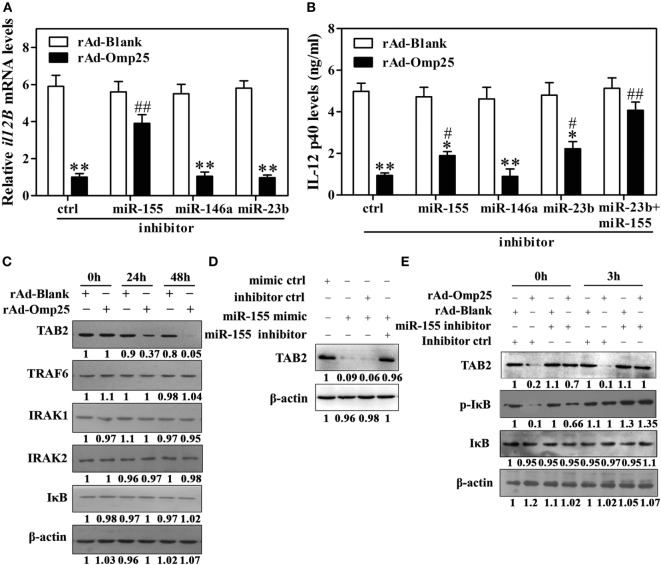
Omp25-induced miR-155 expression inhibits the transcription of *il12B via* targeting to TAB2. **(A,B)** THP-1 cells were infected with recombinant adenoviruses expressing Omp25 protein (rAd-Omp25) or rAd-Blank and transfected with miR-155, miR-146a or miR-23b inhibitor for 24 h, and cell were then incubated with lipopolysaccharide (LPS)/R848 for another 6 or 24 h, and the *il12B* mRNA and interleukin-12 p40 levels were examined by quantitative polymerase chain reaction and ELISA, respectively. **(C)** THP-1 cells were infected with rAd-Omp25 or rAd-Blank for 24 or 48 h, then lysed and analyzed by western blotting. **(D)** THP-1 cells were transfected with mimics control (ctrl), miR-155 mimics or miR-155 mimics and miR-155 inhibitor and treated with LPS/R848 for 24 h. Cell lysates were analyzed by western blotting to detect TAB2. **(E)** THP-1 cells were infected with rAd-Omp25 or rAd-Blank and transfected with miR-155 inhibitor or inhibitor control for 24 h, and cells were then treated with LPS/R848 for another 3 h. Cells were harvested for western blot analysis of TAB2, p-IκB, and IκB levels. The results are means ± SEMs of three independent experiments. *P < 0.05, **P < 0.01 versus rAd-Blank infected cells with same miRNA inhibitors. ^#^*P* < 0.05, ^##^*P* < 0.01 versus rAd-Omp25 infected cells with inhibitor control.

MiR-155 is an NF-κB transactivational target and involved in a negative feedback loop through downregulation of TAB2 or other genes. Our data suggested that miR-155 might be an additional regulator at the transcriptional level in rAd-Omp25-infected cells. We thus hypothesized that miR-155 likely plays a role in Omp25’s inhibition of IL-12 p40 transcription by targeting some key molecules involved in the activation of NF-κB pathway. Indeed, we found that miR-155 inhibitor markedly decreased the suppression of Omp25 to the transcription of *il12B* compared with the inhibitor control, but miR-146a and miR-23b inhibitors did not affect the transcription of *il12B* (Figure 6A). Consequently, miR-155 inhibitor attenuated the suppression of Omp25 on IL-12 p40 production, and together with miR-23b inhibitor, it further blocked the inhibitory effects of Omp25 on the production of IL-12 p40 in THP-1 cells (Figure 6B). We also found that TAB2 was decreased in rAd-Omp25-infected cells compared with rAd-Blank-infected cells, but no change was observed for TRAF6, IRAK1, IRAK2, or IκB (Figure 6C). MiR-155 mimics decreased the level of TAB2 protein expression, suggesting that TAB2 is the target of miR-155 in THP-1 cells (Figure 6D). To further identify the regulatory roles of miR-155 in TAB2 in Omp25-expressed cells, cells were transfected with miR-155 inhibitors or an inhibitor control and infected with rAd-Omp25 or rAd-Blank, followed by stimulation with LPS/R848 for 0 and 3 h. In the presence of inhibitor control, the levels of phospho-IκB and TAB2 were decreased in rAd-Omp25-infected cells compared with rAd-Blank-infected cells, but the reduction of phospho-IκB and TAB2 were partly reversed in the presence of miR-155 inhibitor (Figure 6E). These results suggested that Omp25-induced miR-155 inhibits the transcription of *il12B via* targeting TAB2, and downregulation of miR-155 and -23b can block the suppression of Omp25 on IL-12 p40 expression at transcriptional and posttranscriptional levels, likely by targeting TAB2 and *il12B* 3′UTR, respectively.

### Wild-type *B. suis* Induces Higher Levels of miR-21-5p, miR-23b and miR-155 than the Omp25-Deficient Mutant of *B. suis*

**Figure 7:**
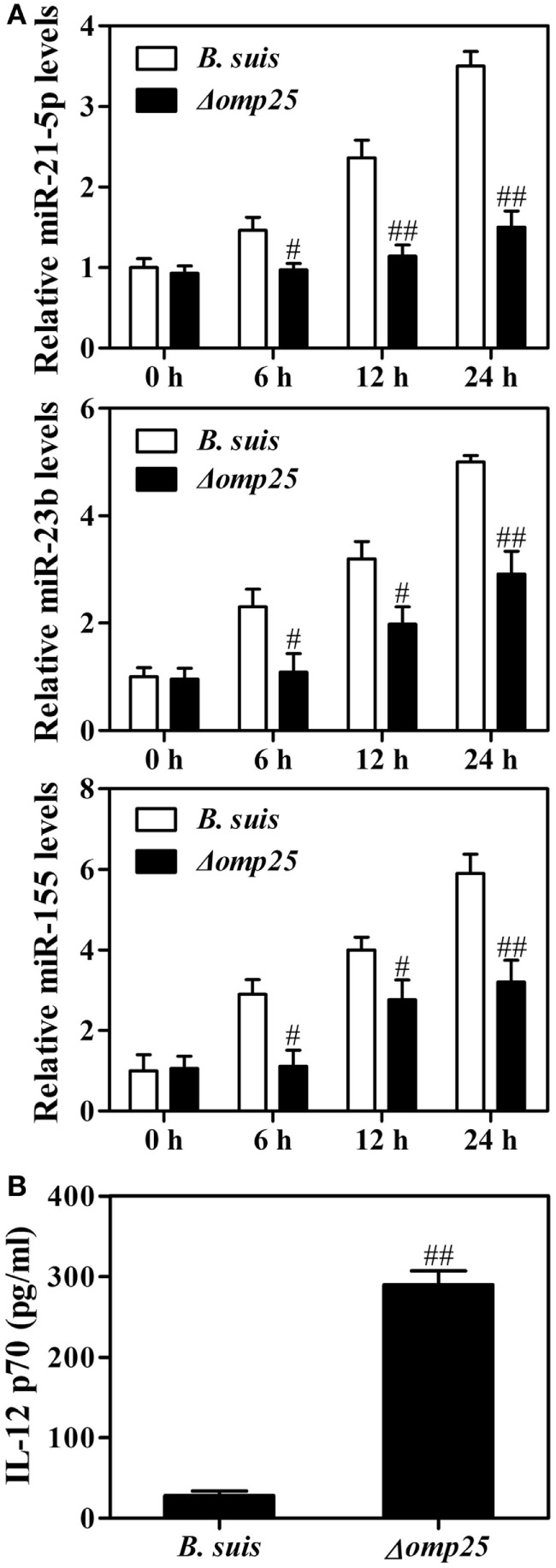
Wild-type (WT) *Brucella suis* induces higher levels of miR-21-5p, miR-23b and miR-155 compared to Omp25-deficient mutant of *B. suis*. **(A)** Human M/MΦs were infected with WT *B. suis* or Δ*omp25* mutant for 0, 6, 12, and 24 h, and the levels of miR-21-5p, miR-23b and miR-155 were analyzed by quantitative polymerase chain reaction. **(B)** Human M/MΦs were infected with WT *B. suis* or Δ *omp25* mutant for 24 h, and cells were then stimulated with lipopolysaccharide (LPS)/R848 for another 24 h in complete culture medium. The concentrations of interleukin (IL)-12 p70 in cell supernatants were determined by ELISA as stated above. The results are means ± SEMs of three independent experiments. ^#^*P* < 0.05, ^##^*P* < 0.01 versus WT *B. suis*-infected cells at same time point.

Now that Omp25 can directly induce miR-21-5p, miR-23b and miR-155 in monocytic THP-1 cells, we suspected if WT *B. suis* could induce a higher level of these miRNAs than the Omp25-deficient mutant. Indeed, we found that the levels of miR-21-5p, miR-23b and miR-155 were higher in the human M/MΦs infected with WT *B. suis* than that in the M/MΦs infected with Omp25-deficient mutant (Δ*omp25 B. suis*) (Figure 7A). Upon stimulation with LPS/R848, Δ*omp25 B. suis*-infected human M/MΦs produced more IL-12 than WT *B. suis*-infected human M/MΦs (Figure 7B). These results further demonstrated that Omp25 plays a key role in the dysfunction of *Brucella*-infected monocyte/macrophages.

### Omp25-Induced PD-1 Expression Is Inversely Associated with IL-12 Production in Human Monocyte/Macrophages

**Figure 8:**
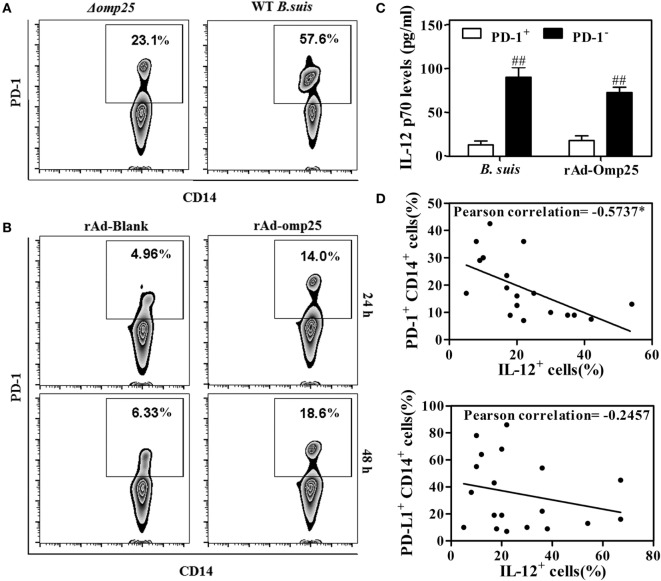
Omp25-induced programmed death-1 (PD-1) expression is inversely associated with interleukin (IL)-12 production in human M/MΦs. **(A,B)** M/MΦs were infected with wild-type (WT) *Brucella suis*, Δ*omp25 B. suis*, recombinant adenoviruses expressing Omp25 protein (rAd-Omp25), or rAd-Blank, and PD-1^+^ CD14^+^ M/MΦs were analyzed by flow cytometry. **(C)** The M/MΦs were infected with WT *B. suis* or rAd-Omp25, PD-1 positive and negative cells were isolated and further stimulated with lipopolysaccharide (LPS)/R848. The levels of IL-12 p70 in cell supernatants were determined by ELISA. ^##^*P* < 0.01 versus PD-1^+^ cells. **(D,E)** M/MΦs derived from different healthy donors were infected with different doses of *B. suis*, followed by LPS/R848 stimulation. The expression of PD-1/PD-L1 and IL-12 were analyzed, and their associations were evaluated using Pearson correlation analysis as shown by negative line trends. *P < 0.05 demonstrated that PD-1 expression is inversely associated with IL-12 production.

Several studies have showed that PD-1 is upregulated in monocyte/macrophages and associated with dysfunction during different infections (30, 31). To determine whether PD-1 is also upregulated and involved in the regulation of IL-12 production in M/MΦs during *Brucella* infection, we examined PD-1 expression on the M/MΦs infected with WT or Δ*omp25 B. suis*, or on the M/MΦs infected with rAd-Omp25 or rAd-Blank. Results showed more PD-1 positive CD14^+^ M/MΦs in WT *B. suis*-infected cells compared to that of Δ*omp25 B. suis-*infected cells (Figure 8A). Similarly, the percentage of PD-1^+^ cells in rAd-Omp25-infected M/MΦs was higher than that in rAd-Blank-infected cells (Figure 8B). In contrast to the suppression of IL-12 production in PD-1^+^ cells during LPS/R848 stimulation, the IL-12 p70 production of PD-1^−^ cells was significantly greater (Figure 8C). Notably, Omp25 significantly increased PD-1 expression in M/MΦs, as well as decreased of IL-12 production.

To determine whether the upregulated PD-1 expression on the surface of M/MΦs correlated with intracellular IL-12 production during *Brucella* infection, M/MΦs derived from different donors were infected with different doses of *Brucella* and stimulated with LPS/R848, then the expression of PD-1, PD-L1, and IL-12 were determined and compared by Pearson correlation analysis. As shown in Figure 8D, the upregulation of PD-1 in M/MΦs was inversely associated with IL-12 production (*r* = −0.5737, *P* = 0.0102) (representative dot plot experiment in inset). Interestingly, although the expression of PD-L1 was also upregulated in infected M/MΦs, it did not significantly correlate with IL-12 production (Figure 8E).

### Blocking PD-1 Signaling Decreases miRNAs Levels and Enhances IL-12 Production

**Figure 9:**
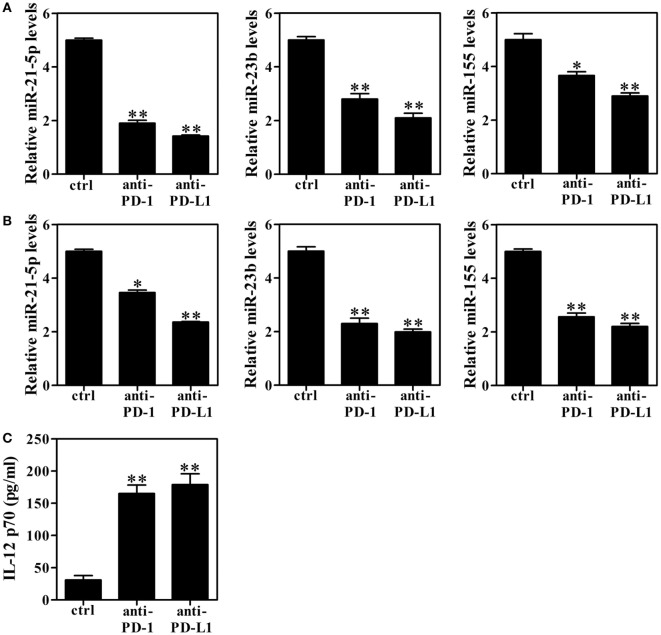
Blocking programmed death-1 (PD-1) signaling decreases miRNAs levels and enhances interleukin (IL)-12 production. **(A,B)** THP-1 cells and M/MΦs isolated from healthy donors were infected with wild-type *Brucella suis* and preincubated with anti-PD-1, anti-PD-L1, or control IgG Abs (ctrl) overnight and then stimulated with lipopolysaccharide (LPS)/R848. Real-time PCR was used to detect the relative changes of miR-21-5p, miR-23b and miR-155 in THP-1 cells **(A)** and M/MΦs **(B)**. **(C)** The levels of IL-12 p70 in M/MΦs were detected with ELISA. *P < 0.05, **P < 0.01 versus control IgG Abs (ctrl).

Because PD-1^+^ cells displayed impaired IL-12 production, we next sought to define the relationship between PD-1 and miRNAs (miR-21-5p, miR-23b and miR-155) upregulation as a potential mechanism underlying Omp25-mediated IL-12 inhibition. As an initial approach, we investigated whether blockade of the PD-1 pathway affected Omp25-induced miRNAs expression in M/MΦs. THP-1 cells and M/MΦs isolated from healthy donors were infected with WT *B. suis* and preincubated with anti-PD-1, anti-PD-L1, or control IgG Abs overnight followed by stimulation with LPS/R848. Incubation of THP-1 cells or healthy M/MΦs with anti-PD-1 or anti-PD-L1 Ab resulted in significant suppression of miR-21-5p, miR-23b and miR-155 compared with the control IgG (Figures 9A,B). Omp25-induced IL-12 inhibition was also reduced by either anti-PD-1 or anti-PD-L1 treatment (Figure 9C). Together, these data suggested that modulation of PD-1 signaling can significantly improve the dysfunction of *Brucella*-infected M/MΦs and promote IL-12 production.

## Discussion

**Figure 10:**
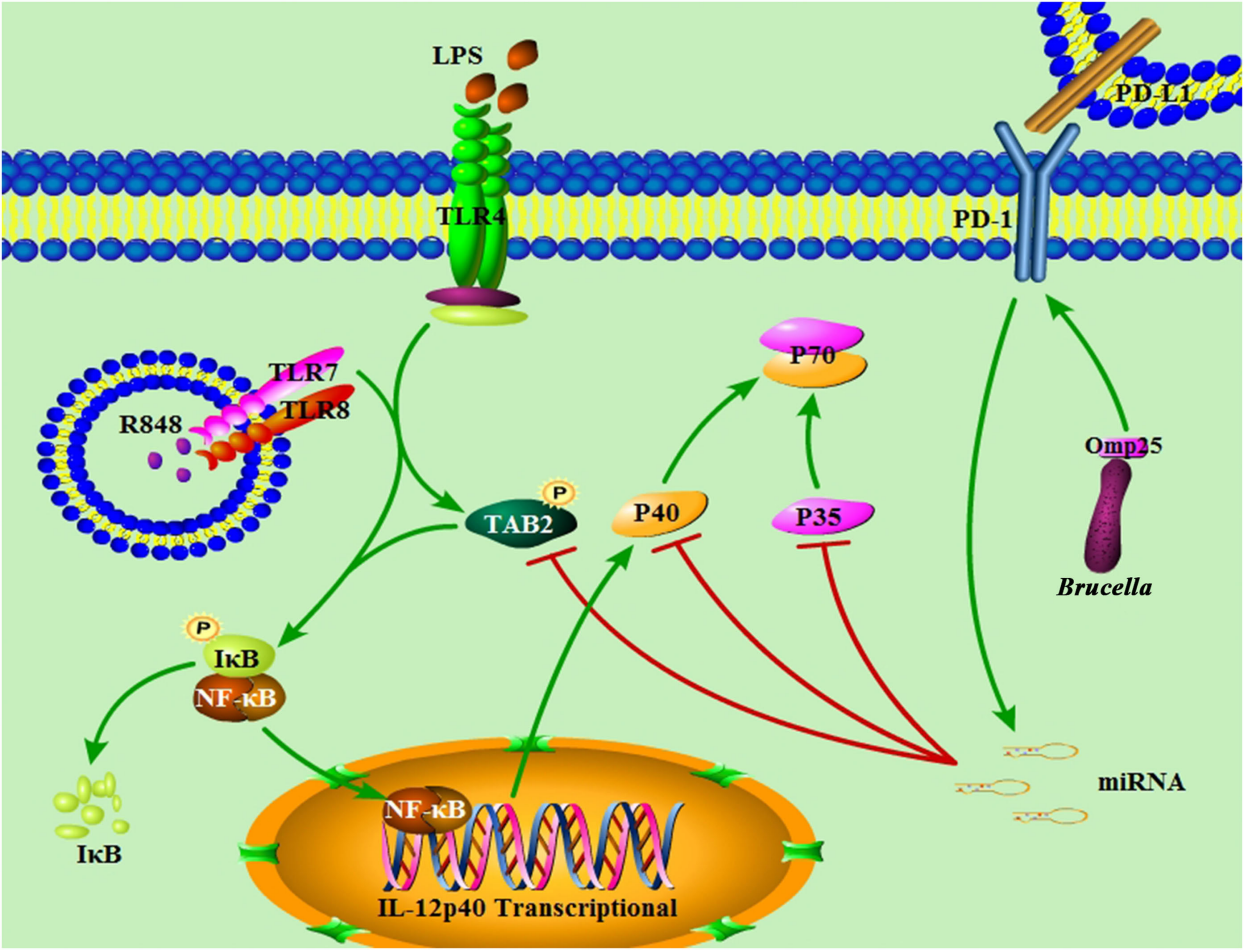
Model of *Brucella* Omp25-mediated inhibition of interleukin (IL)-12 production in human monocyte/macrophages. *Brucella* Omp25 inhibits TLR agonist-induced IL-12 p35 and p40 subunits at both the transcriptional and posttranscriptional levels *via* activating programmed death-1 (PD-1) signaling and upregulating miR-21-5p, miR-23b and miR-155, together resulting in a decreased IL-12 production in human monocyte/macrophages.

Despite extensive work in the field of *Brucella*-induced innate and adaptive immunity dysfunction (5, 20, 32, 33), the molecular mechanisms of Omp25-mediated monocyte/macrophages dysfunction, including the potential regulatory role of miRNA involved in this process, remains incompletely understood. In this study, we demonstrated the important roles and the underlying mechanisms of *Brucella* Omp25 in inhibiting TLR agonists-induced IL-12 production in monocyte/macrophages. In Omp25-expressing cells, the upregulation miR-21-5p, miR-23b, miR-155 and PD-1 correlated with decreased IL-12 p35 and p40 levels, resulting in a decreased IL-12 p70 production following LPS/R848 stimulation. In this process, miR-21-5p and miR-23b appeared to directly target the 3′UTR of *il12A* and *il12B* that downregulated the expression of IL-12 p35 and p40 subunits at the posttranscriptional level, while miR-155 directly targets the 3′ UTR of TAB2 to downregulate the expression of IL-12 p40 subunit at the transcriptional level *via* inhibiting the activation of NF-κB pathway (Figure 10). Consistently, WT *B. suis*-infected M/MΦs exhibited an increased level of PD-1 expression and induced higher levels of miR-21-5p, miR-23b and miR-155 than the Δ*omp25 B. suis mutant*, which correlated with the degree of IL-12 inhibition. By contrast, blocking PD-1 signaling decreased miRNAs levels and further enhanced IL-12 production.

MicroRNAs are regulators of inflammation in various cell types, including monocyte/macrophages (33, 34). Numerous miRNAs have been found to be upregulated or downregulated in pathogen-infected or in activated immune cells. In *B. melitensis*-infected mouse macrophage cell line RAW264.7 cells, microRNA expression profile identified 57 differentially expressed miRNAs between mock- and *Brucella*-infected cells (35). MiR-146a is the first miRNA identified to negatively regulate TLR4 signaling in human monocytic cells stimulated with LPS (36). MiR-146a feedback inhibits RIG-I-dependent Type I IFN production in macrophages by targeting tumor necrosis factor receptor-associated factor 6 (TRAF6) and IL-1 receptor–associated kinase 1 and 2 (IRAK1 and IRAK2) (37). MiR-146a is also found to represses mycobacteria-induced inflammatory response and facilitate bacterial replication *via* targeting IRAK1 and TRAF6 (38). However, in *B. melitensis*-infected RAW264.7 cells (35) and Omp25-expressing THP-1 cells, miR-146a is not upregulated, and miR-146a inhibitor does not decrease the suppression of Omp25 to the transcription of *il12B* compared with the inhibitor control. MiR-155 is induced in macrophages in response to both bacterial and viral infection that activates TLR4, TLR2, TLR3, or TLR9 (39, 40). MiR-155 have been shown to target transforming growth factor-β-activated kinase 1-binding protein 2 (TAB2) and Pellino-1, adaptors in the signaling complex that activates IκB kinase β, which suggests that miR-155 is part of a negative feedback loop to dampen inflammatory responses (41). In Omp25-expressing THP-1 cells, we found that miR-155 was upregulated and TAB2 were decreased, while TRAF6, IRAK1, and IRAK2 did not change. Consequently, miR-155 inhibitor decreased the suppression of Omp25 on the transcription of *il12B* compared with the inhibitor control and miR-146a inhibitor. MiR-21-5p has been identified to inhibit IL-12 p35 expression at posttranscriptional level by targeting the 3′UTR of *il12A* in previous studies (42), and miR-23b has been reported to promote the tolerogenic properties of dendritic cells through inhibiting Notch1/NF-kappa B signaling (43). In this study, we found that miR-21-5p and miR-23b were upregulated and correlated with gradually decreasing IL-12 p35 and p40 levels in Omp25-expressing THP-1 cells. Downregulation of miR-23b and miR-21-5p partly blocked Omp25 inhibitory effect on LPS/R848-induced IL-12 p70 production. Our results not only confirmed that miR-21-5p inhibits IL-12 p35 expression at posttranscriptional level by targeting to the 3′ UTR of *il12*A but also found that miR-23b inhibits IL-12 p40 expression at posttranscriptional level by targeting to the 3′ UTR of *il12*B. However, other predicted miRNAs that also target the 3′UTR of *il12*A and *il12*B, including miR-590-5p, miR-340-5p, miR-23a, miR-23c and miR-494-3p, were not involved in the inhibition of IL-12 production in Omp25-expressing THP-1 cells. In addition, our data showed that the expression of miR-21-5p, miR-23b and miR-155 is downregulated in Δ*omp25 B. suis* infection compared with that WT *B. suis* infection. These results demonstrate an important role of these miRNAs in the regulation of IL-12 expression in *Brucella*-infected monocyte/macrophages.

Programmed death-1 signaling has been suggested to be involved in the inhibition of some proinflammatory cytokines production, resulting in compromised Type1 (Th1) cellular immune response (31, 44, 45). However, the roles and molecular mechanism(s) of PD-1-mediated monocyte/macrophages dysfunction have not been defined in *Brucella* infection. In the present study, WT *B. suis* infection and Omp25 expression significantly increased PD-1 expression, which is inversely correlated with IL-12 production in monocyte/macrophages; anti-PD-1 and anti-PD-L1 treatment blocked the IL-12 inhibition induced by WT *B. suis*. Furthermore, we defined the relationship between PD-1 expression and miRNAs (miR-21-5p, miR-23b and miR-155) and demonstrated that PD-1 signaling is critical for upregulation of miR-21-5p, miR-23b and miR-155 in Omp25-expressing cells as well as WT *B. suis-*infected cells.

In summary, in this work, we have provided strong evidence for the roles of microRNAs (miR-155, -23b and -21-5p) and PD-1 signaling in regulating Omp25-induced IL-12 inhibition in *Brucella*-infected monocyte/macrophages and demonstrated the regulation patterns of different molecules in this process. Our findings would help further understand the mechanism(s) of *Brucella*-induced monocyte/macrophages dysfunction and provide new targets for the development of novel therapeutic or vaccination approaches.

## Author Contributions

YH, DT, BC, and S-LL designed the experiments and wrote the paper. BC, WL, YC, XW, QD, and XZ performed the experiments. YH, BC, and HZ analyzed the data. WL contributed the human blood. YH, DT, and HZ contributed reagents/materials/analysis tools.

## Conflict of Interest Statement

The authors declare that the research was conducted in the absence of any commercial or financial relationships that could be construed as a potential conflict of interest.

## Footnotes

^1^http://www.mirbase.org.

^2^http://www.ncbi.nlm.nih.gov/.
