# Supplementary material for: Brucella Omp25 Upregulates miR-155, miR-21-5p, and miR-23b to Inhibit Interleukin-12 Production via Modulation of Programmed Death-1 Signaling in Human Monocyte/Macrophages
Source: Front Immunol. 2017 Jun 26;8:708. doi: 10.3389/fimmu.2017.00708 (PMC5483987; doi:10.3389/fimmu.2017.00708)
Supplement: Supplementary file 1 [file Data_Sheet_2.doc]

**Supplementary Material**

**
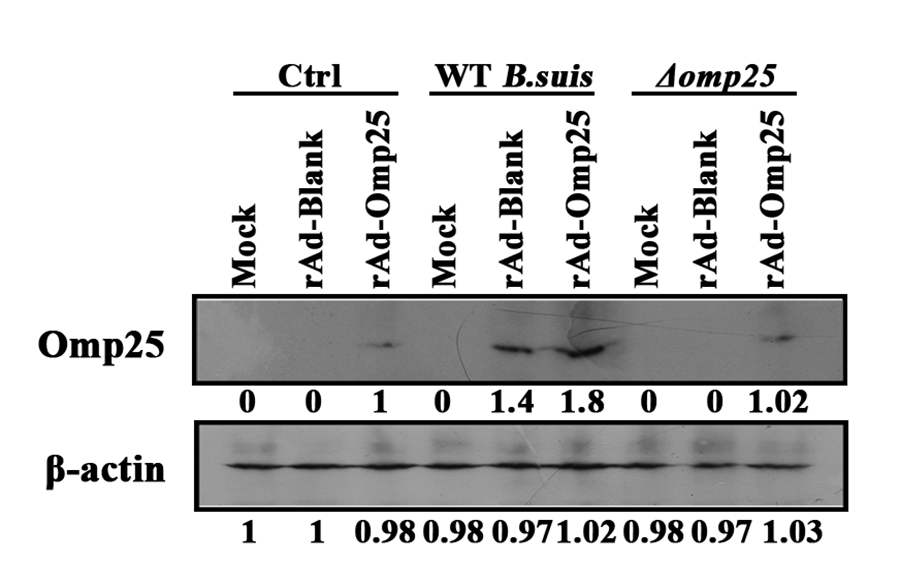
**

**Figure S1. Expression of Omp25 in *wild-type B. suis* and *Δomp25 B. suis*-infected cells.** THP-1 cells were infected with 100 MOI of rAd-Omp25 or rAd-Blank for 24 h, and then infected with *WT B. suis* or *Δomp25 B. suis* for another 24 h or uninfected (Ctrl) as described in Methods, followed by western blotting detection of Omp25.


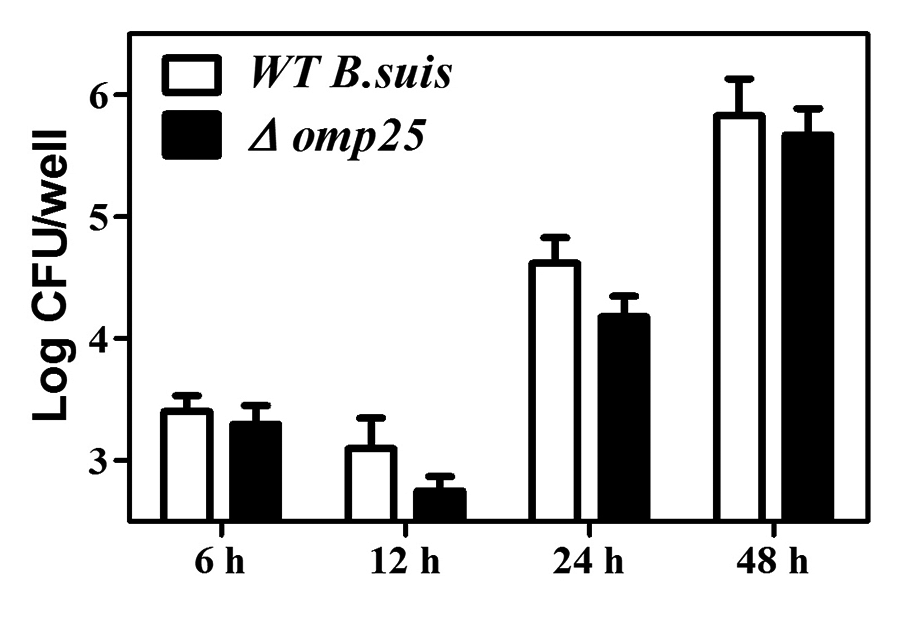


**Figure S2. The intracellular survival of *wild-type B. suis* and *Δomp25 B. suis.*** THP-1 cells were infected with *WT B. suis* or *Δomp25 B. suis*. At 6, 12, 24 and 48 h p.i., the numbers of viable intracellular bacteria were determined as described in Methods. The results are means ± SEMs of 3 independent experiments.


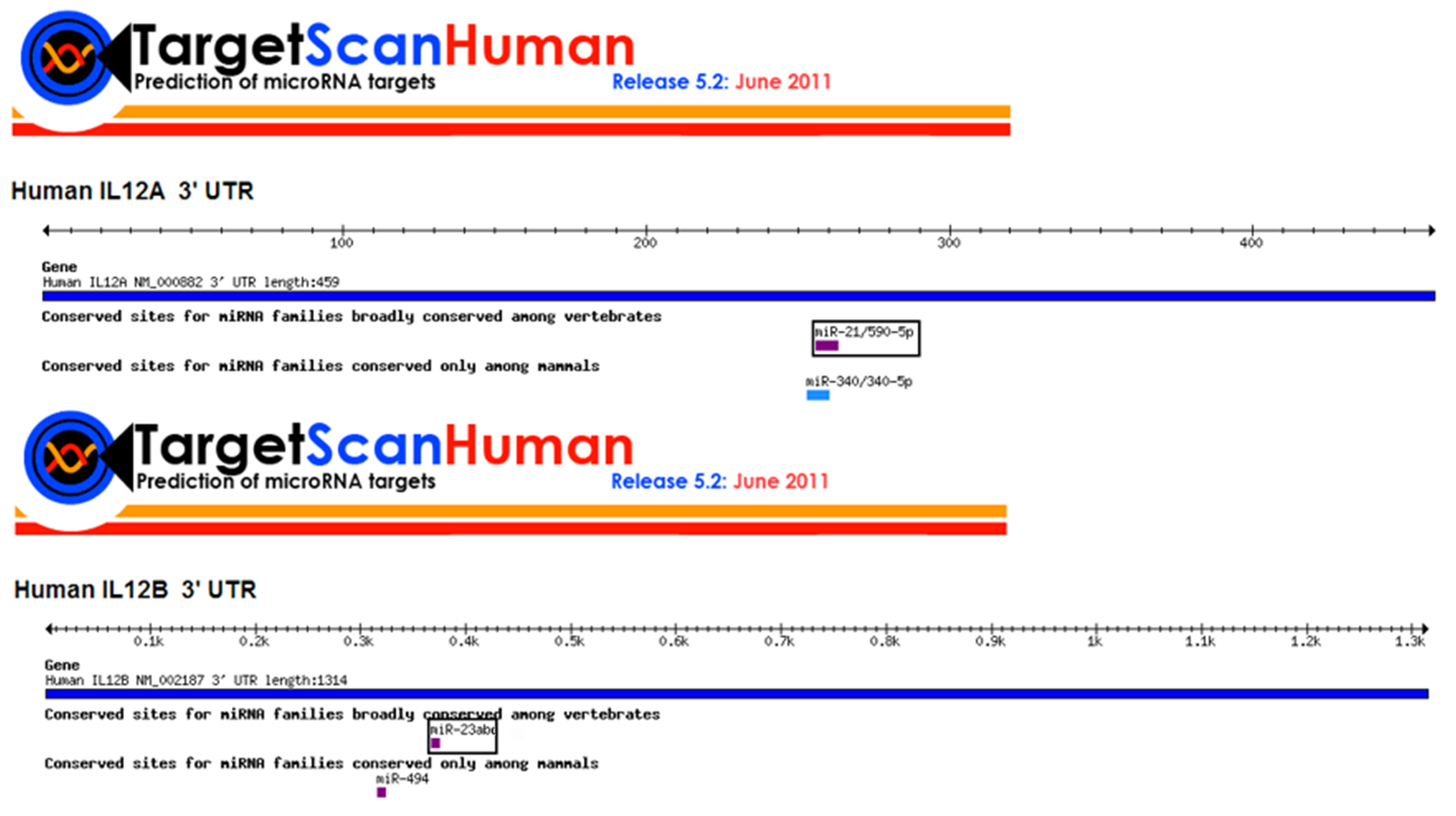


**Figure S3.** **Predicted miRNA recognition elements in the 3’ UTR regions of *il12A* and *il12B* genes.** MiRNA prediction algorithms were used to evaluate the likelihood of miRNA binding in the 3’ UTR regions of *il12A* and *il12B* genes.


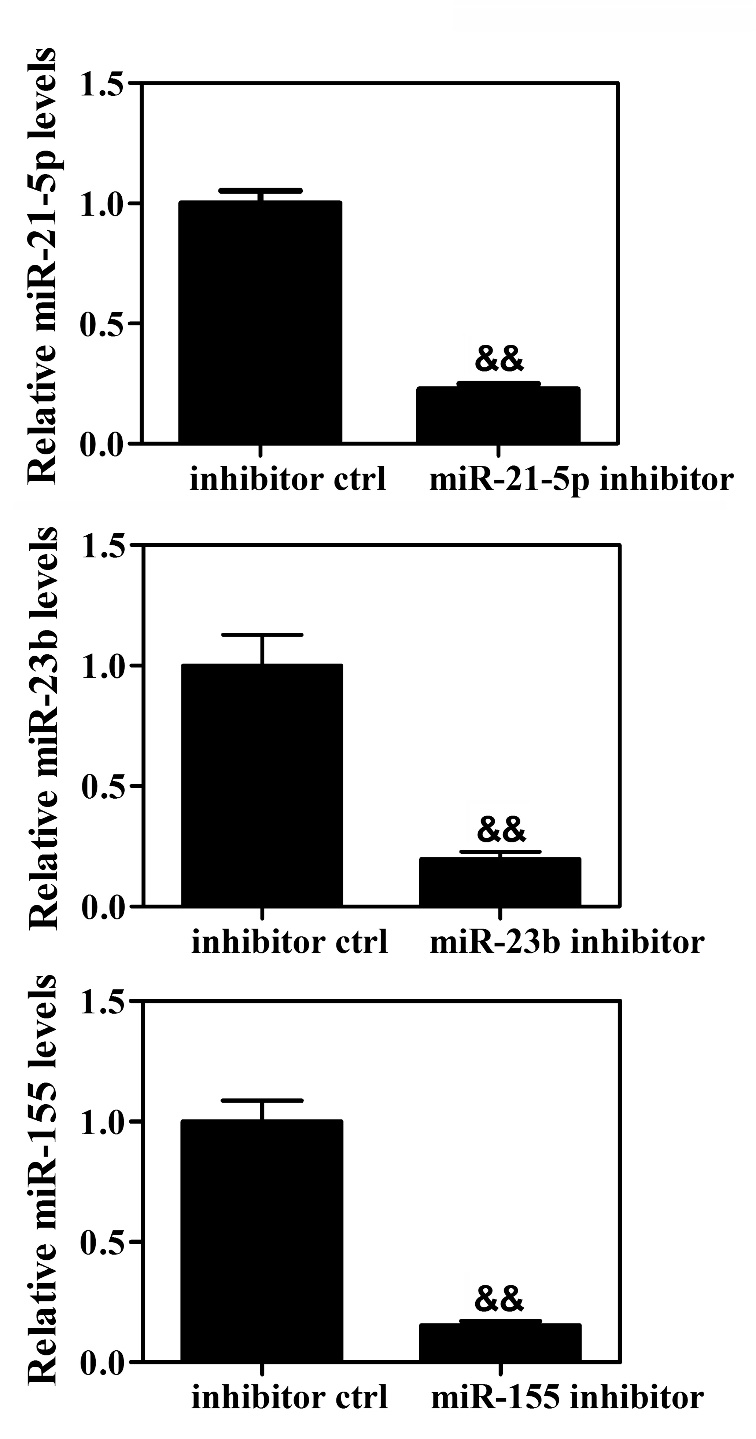


**Figure S4. The effects of miRNA inhibitors.** The rAd-Omp25-infected THP-1 cells were respectively treated with miR-21-5p, miR-23b or miR-155 inhibitor for 24 h, and the corresponding miRNAs were mesasured by Q-PCR and normalized to the expression of RNU6B in each sample. The results are mean ± SEM of 3 independent experiments. ^&&^*P* < 0.01 versus cells transfected inhibitor control.


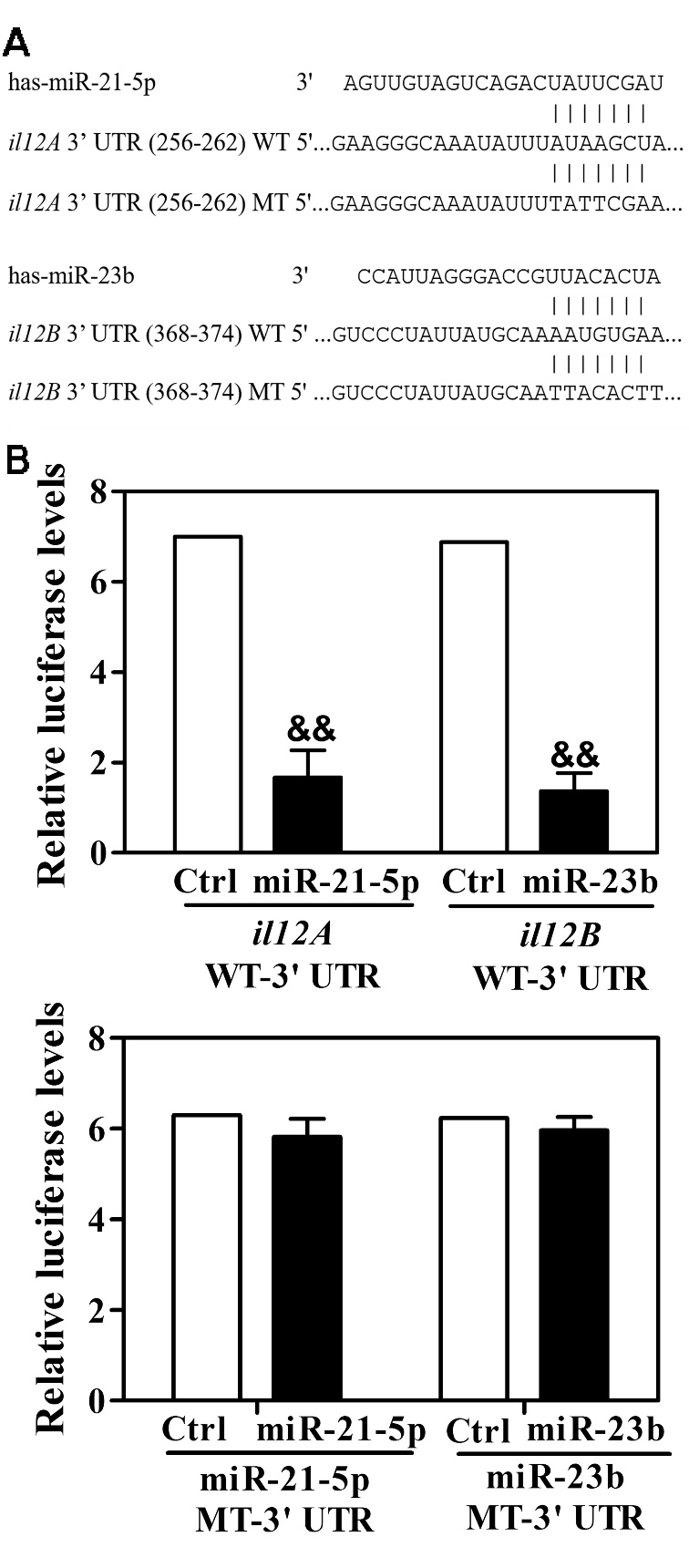


**Figure S5. Omp25-induced miR-21-5p and miR-23b inhibit the expression of IL-12 p35 and p40 in the post-transcriptional level respectively. (A)** Human *il12A* and *il12B* might be the molecular target of miR-21-5p and miR-23b respectively. This diagram represents a sequence alignment of miR-21-5p or -23b, and their target sites in the 3’ UTR regions of *il12A* and *il12B* genes, and relative mutated versions. **(B)** MiRNAs reduced luciferase activity in cells transfected with wild-type reporter (*il12A* or *il12B* WT-3’ UTR), but not in cells transfected with mutated-type reporter (miR-21-5p or 23b MT-3’ UTR). HEK-293 cells were transfected with wild (or mutated) type *il12A* or *il12B* 3’ UTR firefly luciferase reporter plasmids, pTK-Renilla-luciferase plasmids, together with control (ctrl), miR-21-5p or miR-23b. After 48 hours, firefly luciferase activity was measured and normalized by renilla luciferase activity. The results are mean ± SEM of 3 independent experiments. ^&&^*P* < 0.01 versus cells transfected control mimics.

**Table S1 Primers used in this study.**

| **Primer ID** | | **Primer sequence (5’-3’)** |
| --- | --- | --- |
| Omp25 FW | | CCGCTCGAGACCATGCGCACTCTTAAGTCTCT |
| Flag-Omp25RW | | AAGATATCTTACTTGTCGTCATCGTCTTTGTAGTCGAACTTGTAGCCGATGCC |
| HM21 RT primer | | GTTGGCTCTGGTGCAGGGTCCGAGGTATTCGCACCAGAGCCAACTCAACA |
| HM21 FW | | CGGCGTAGCTTATCAGACTGA |
| HM590 RT primer | | GTTGGCTCTGGTGCAGGGTCCGAGGTATTCGCACCAGAGCCAACCTGCAC |
| HM590 FW | | CGGCGGAGCTTATTCATAAAAG |
| HM23a RT primer | | GTTGGCTCTGGTGCAGGGTCCGAGGTATTCGCACCAGAGCCAACGGAAAT |
| HM23a FW | | CTGATCACATTGCCAGGG |
| HM23b RT primer | | GTTGGCTCTGGTGCAGGGTCCGAGGTATTCGCACCAGAGCCAACTGGTAA |
| HM23b FW | | CTGATCACATTGCCAGGGA |
| HM23c RT primer | | GTTGGCTCTGGTGCAGGGTCCGAGGTATTCGCACCAGAGCCAACGGGTAA |
| HM23c FW | | CTGATCACATTGCCAGTGA |
| HM494 RT primer | | GTTGGCTCTGGTGCAGGGTCCGAGGTATTCGCACCAGAGCCAACGAGGTT |
| HM494 FW | | GGCGCTGAAACATACACGGGA |
| HM146a RT primer | | GTTGGCTCTGGTGCAGGGTCCGAGGTATTCGCACCAGAGCCAACAACCCA |
| HM146a FW | | CGGCTGAGAACTGAATTCCA |
| HM146b RT primer | | GTTGGCTCTGGTGCAGGGTCCGAGGTATTCGCACCAGAGCCAACAGCCTA |
| HM146b FW | | GGCGCTGAGAACTGAATTCCA |
| HM155 RT primer | | GTTGGCTCTGGTGCAGGGTCCGAGGTATTCGCACCAGAGCCAACACCCCTA |
| HM155 FW | | CGGCTTAATGCTAATTGTGA |
| RNU6B RT primer | | GTTGGCTCTGGTGCAGGGTCCGAGGTATTCGCACCAGAGCCAACAAAAATAT |
| RNU6B FW | | TTCCTCCGCAAGGATGACACGC |
| Reverse primer | | GTGCAGGGTCCGAGGT |
| IL-12 p40_qPCR_F | | GTGAGGTCTTAGGCTCTGGC |
| IL-12 p40_qPCR_R | | AACCTCGCCTCCTTTGTGAC |
| IL-12 p35_qPCR_F | | CTCCTCCTTGTGGCTACCCT |
| IL-12 p35_qPCR_R | | AGGCATGGGAACATTCCTGG |
| β-actin_qPCR_F | | GCGCGGCTACAGCTTCACCA |
| β-actin_qPCR_R | | GGGCAGCGGAACCGCTCATT |
| *il12A*-Promotor_F | | GGGGTACCCATGACTGCGCTTCTGATCCC |
| *il12A*-Promotor_R | | CCCAAGCTTGTGCCCGGCCGCAGCCGTT |
| *il12B*-Promotor_F | | GGGGTACCCTGTATGCCTCCCTGAGGGTAT |
| *il12B*-Promotor_R | | CCCAAGCTTAGTGCTTACCTTGCTCTGGG |
| *il12A-*WT-3’UTR_F | | CGACGCGTAAAAGCGAGGTCCCTCCAA |
| *il12A-*WT-3’UTR_R | | CGAGCTCTTTTTTTTCACTTTAATTCAATACT |
| *il12A-*MT-3’UTR_F1 | | CGACGCGTAAAAGCGAGGTCCCTCCAA |
| *il12A-*MT-3’UTR_R1 | | TCGCCTAAAATATTTGCCCT |
| *il12A-*MT-3’UTR_F2 | | TATTCGAATTTCTGTACCAAAG |
| *il12A-*MT-3’UTR_R2 | | CGAGCTCTTTTTTTTCACTTTAATTCAATACT |
| *il12B-*WT-3’UTR_F | | CGACGCGTGTTCTGATCCAGGATGAAA |
| *il12B-*WT-3’UTR_R | | CGAGCTCGATTACAAAGAAGAGTTTTTATTAG |
| *il12B-*MT-3’UTR_F1 | | CG ACGCGTGTTCTGATCCAGGATGAAA |
| *il12B-*MT-3’UTR_R1 | | AGTGTAATTGCATAATAGGGA |
| *il12B-*MT-3’UTR_F2 | | TTACACTATTTAATTTTATTTGTACTG |
| *il12B-*MT-3’UTR_R2 | | CGAGCTCGATTACAAAGAAGAGTTTTTATTAG |
|  |  | |
